# Inhalable vaccine of bacterial culture supernatant extract mediates protection against fatal pulmonary anthrax

**DOI:** 10.1080/22221751.2023.2191741

**Published:** 2023-03-31

**Authors:** Li-Na Zhai, Yue Zhao, Xiao-Lin Song, Tong-Tong Qin, Zhi-Jun Zhang, Jia-Zhen Wang, Cheng-Yu Sui, Li-Li Zhang, Meng Lv, Ling-Fei Hu, Dong-Sheng Zhou, Tong-Yu Fang, Wen-Hui Yang, Yan-Chun Wang

**Affiliations:** a College of Life Science and Technology, Beijing University of Chemical Technology, Beijing, People’s Republic of China; b State Key Laboratory of Pathogen and Biosecurity, Beijing Institute of Microbiology and Epidemiology, Beijing, People’s Republic of China; c State Key Laboratory of Pathogens and Biosecurity, Beijing Institute of Biotechnology, Beijing, People’s Republic of China; d Basic medical college, Guizhou Medical University, Guizhou, People’s Republic of China

**Keywords:** Inhalable vaccine, pulmonary anthrax, culture supernatant extract, protective antigen, mucosal immune response

## Abstract

Pulmonary anthrax is the most fatal clinical form of anthrax and currently available injectable vaccines do not provide adequate protection against it. Hence, next-generation vaccines that effectively induce immunity against pulmonary anthrax are urgently needed. In the present study, we prepared an attenuated and low protease activity *Bacillus anthracis* strain A16R-5.1 by deleting five of its extracellular protease activity-associated genes and its *lef* gene through the CRISPR-Cas9 genome editing system. This mutant strain was then used to formulate a lethal toxin (LeTx)-free culture supernatant extract (CSE) anthrax vaccine, of which half was protective antigen (PA). We generated liquid, powder, and powder reconstituted formulations that could be delivered by aerosolized intratracheal inoculation. All of them induced strong humoral, cellular, and mucosal immune responses. The vaccines also produced LeTx neutralizing antibodies and conferred full protection against the lethal aerosol challenges of *B. anthracis* Pasteur II spores in mice. Compared to the recombinant PA vaccine, the CSE anthrax vaccine with equal PA content provided superior immunoprotection against pulmonary anthrax. The preceding results suggest that the CSE anthrax vaccine developed herein is suitable and scalable for use in inhalational immunization against pulmonary anthrax.

## Introduction

Anthrax is an acute, virulent infectious disease caused by the Gram-positive, spore-forming, facultative aerobe *Bacillus anthracis* [1]. Pulmonary (inhalational) anthrax is caused by inhaled *B. anthracis* spores that are engulfed by alveolar macrophages in which they bud into rapidly dividing vegetative cells that secrete toxins and virulence factors [2]. This form of anthrax is clinically the most severe and ultimately causes massive bacteremia and toxemia followed by multi-organ failure and septic shock. Untreated, its mortality rate nears 100% [3]. *B. anthracis* spores are extremely resistant and the disease they cause has high lethality. They can infect hundreds of thousands of people via aerosol dissemination and constitute a major biological agent [4].

As *B. anthracis* is highly lethal and could potentially be used as a bioweapon, anthrax vaccines have been developed. One approved human anthrax vaccine is a live attenuated form consisting of nonvirulent spores derived from the STI-1 (Russia) and A16R (China) strains. Nevertheless, their application is limited as they can cause infection and their injection may trigger adverse reactions [5, 6]. Another approved human anthrax vaccine is a cell-free culture supernatant with protective antigen (PA) as its main component. Its two main forms are anthrax vaccine adsorbed (AVA, USA) and anthrax vaccine precipitated (AVP, UK). However, repeated subcutaneous annual injections and booster doses can induce local and systemic side effects [7]. Moreover, prior research has demonstrated that only AVA provided complete protection against fatal pulmonary anthrax.

Researchers have focused on next-generation anthrax vaccines as these could be safer and more effective than their predecessors. PA is the core antigen in recent vaccine designs [8]. It binds the lethal factor (LF, a Zn^2+^-dependent protease) and edema factor (EF, adenylyl cyclase) to form lethal toxin (LeTx) and edema toxin (ET) [9], and mediates the penetration of the two toxins into host target cells, thereby causing cellular toxicity and lethality. In past decades, various recombinant PA (rPA)-based vaccines have been developed and some of them are currently being validated in clinical trials. They appear to be safe, immunogenic, and relatively simple to manufacture. Nevertheless, it has not yet been empirically or clinically established whether their performance is at least comparable to that of AVA.

Ongoing efforts to enhance the efficacy of rPA-based vaccines have included combining them with other antigens, adding adjuvants to them, or changing their administration routes [10]. We previously prepared dry powder and liquid inhalations by adding CpG oligonucleotide (CpG) adjuvant based on rPA. Mice vaccinated with either rPA vaccine formulation by aerosolized intratracheal (i.t.) inoculation were fully protected against a 20 × LD_50_ aerosol *B. anthracis* spore challenge [11]. Meanwhile, another study demonstrated that a combination of subcutaneous inoculation of rPA and *B. anthracis* spores provided relatively better protection against lethal-dose *B. anthracis* aerosol exposure than rPA alone in guinea pigs, but neither treatment conferred full protection [12]. Despite there being methodological differences between the aforementioned studies, we nonetheless hypothesize that the i.t. route has advantages over the subcutaneous (s.c.) route and will be the future direction of vaccine development, though the vaccine compositon could be further optimized to against higher-doses *B. anthracis* spores challenge.

AVA and the vaccines derived from it have proven effective at preventing pulmonary anthrax [13, 14]. The *B. anthracis* V770-NP1-R strain used in AVA preparation is unencapsulated and has low protease activity [15]. Our previous work demonstrated that PA was upregulated and its degradation was inhibited in a mutant *nprR*-deficient *B. anthracis* strain [16]. Therefore, we hypothesized that a reduction in protease activity could mitigate the degradation of multiple known and unknown antigens secreted by the vaccine strain. Furthermore, certain extracellular proteases such as LF are virulence factors affecting vaccine safety [17]. Hence, a promising molecular design strategy is to knock out as many protease activity-associated genes as possible to augment immunogenicity, attenuate the strain, and prepare next-generation cell-free culture supernatant vaccines.

Based on the foregoing discoveries, we generated culture supernatant extract (CSE) anthrax vaccines based on a strain with deletion of five proteases activity-associated genes. We then administered CSE-based vaccines with various formulations and adjuvants to mice via different vaccination routes and evaluated the safety, immunogenicity, and protective efficacy of these vaccines against pulmonary anthrax. We then compared CSE and rPA to establish the relative differences in their protective effects. The results of the present study preclinically demonstrated the feasibility of a novel culture supernatant extract anthrax vaccine administered by the aerosolized i.t. route.

## Materials and methods

### Bacterial strains and culture conditions

All bacterial strains used in the present study are listed in **Table S1**. The initial strain was attenuated *B. anthracis* A16R (pXO1^+^, pXO2^−^). All *B. anthracis* derivatives were prepared in the laboratory and stored at – 80 °C. The strains were warmed at 20 − 25 °C, resuscitated by the antibiotic-free Luria–Bertani (LB, BD Biosciences, Franklin Lakes, NJ, USA) solid culture plate streaking method, and placed at 37 °C. By the following day, a single colony was inoculated onto 5 mL brain heart infusion (BHI, BD Biosciences, Franklin Lakes, NJ, USA) medium and shaken at 220 rpm, 37 °C for 9 h. The bacterial suspensions were inoculated onto LB solid medium and placed overnight in an incubator at 37 °C. All colonies were scraped off the plate and transferred to RM base medium [18] (Coolaber, Beijing, China) at a rate of 100 µL overnight culture/100 mL and incubated with shaking at 110 rpm, 37 °C for 12 h. For the subsequent challenge assays, *B. anthracis* Pasteur II strain spores were prepared according to a previously protocol [11].

### Mutant strain construction and characterization for vaccine preparation

**Figure 1.**
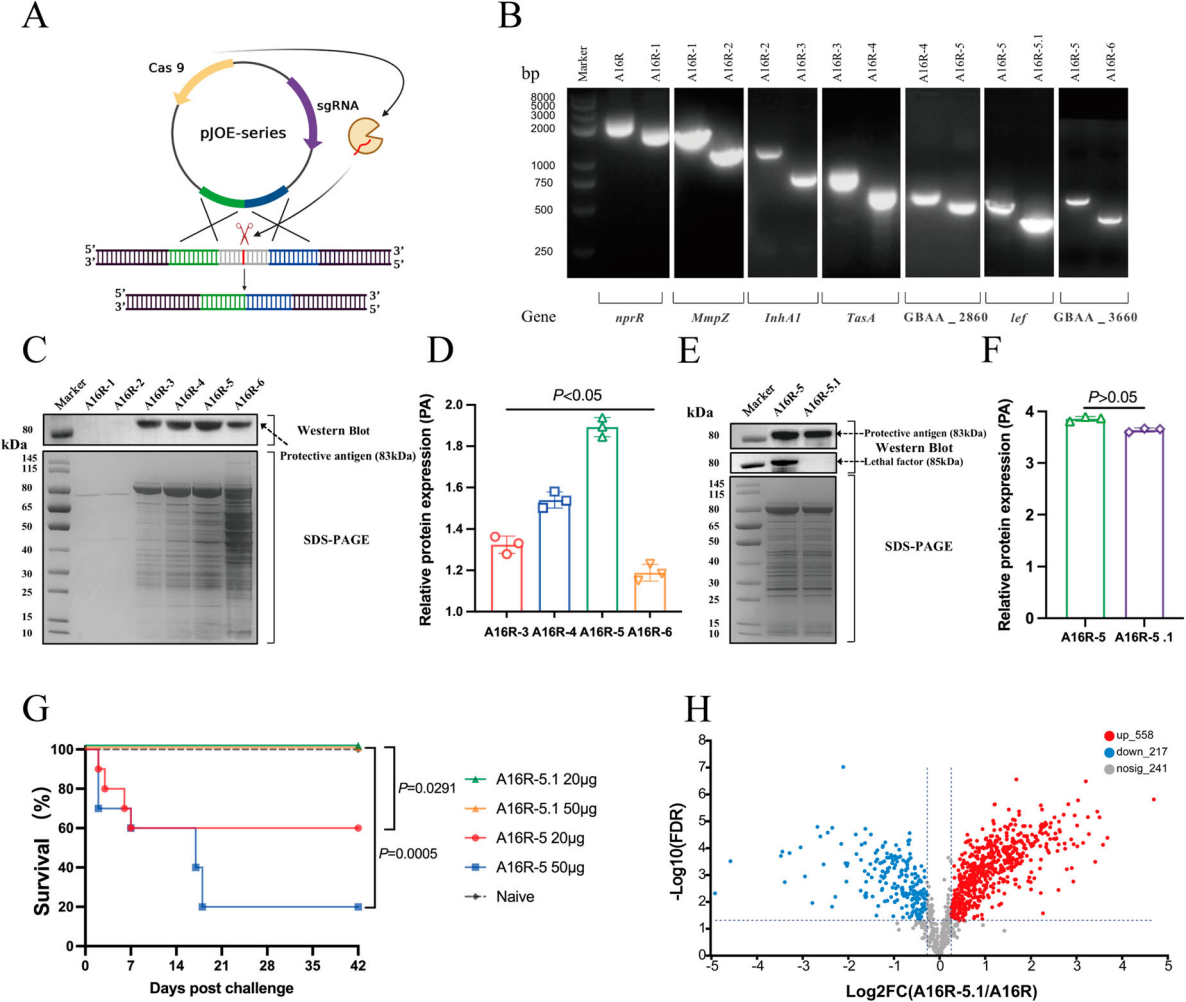
Characterization of *B. anthracis* mutants deficient in extracellular protease activity. **(A)** Schematic diagram of CRISPR-Cas9 genome editing in *B. anthracis*. **(B)** PCR identification of *B. anthracis* mutant with reduced proteolytic activity. **(C)** Mutant strains identified by SDS-PAGE and western blot analysis with mouse anti-PA polyclonal antibodies. **(D)** The relative expression levels of PA in the mutant strains analyzed by ImageJ. **(E)** A16R-5 and A16R-5.1 strains identified by SDS-PAGE and western blot analysis with mouse anti-PA and anti-LF polyclonal antibodies. **(F)** The relative expression levels of PA in A16R-5 and A16R-5.1 strains analyzed by ImageJ. **(G)** Survival in mice (n = 10) immunized with A16R-5 and A16R-5.1 via i.t. inoculation. **(H)** Volcano plots of differentially expressed proteins (DEPs) in A16R culture supernatant and CSE vaccine.

All plasmids used in the present study are listed in **Table S1**. The primers used herein are listed in **Table S2**. To obtain the optimal mutant strain for vaccine preparation, multiple extracellular protease activity-related genes (*nprR*, *mmzp*, *inhA1*, *tasA, GBAA_2860, lef,* and *GBAA_3660)* were sequentially deleted using CRISPR (Clustered Regularly Interspaced Palindromic Repeats)-Cas9 genome editing system (Figure 1A) according to previously protocol [19]. The mutant sites were identified by PCR (polymerase chain reaction) using gene-specific primers. The culture supernatants of various *B. anthracis* strains were collected by centrifugation at 8,000 × *g* and 4 °C for 20 min, filtered (431097, 0.22 μm, Corning, NY, USA) to remove residual bacteria, then concentrated in the constant proportion (from 500 mL to 50 mL) by tangential flow (ultra) filtration (Vivaflow 200, Sartorius, Göttingen, Germany) and washed with phosphate-buffered saline (PBS) to remove residual medium. The concentrate was resuspended in 50 mL PBS and ultrafiltered through Vivaspin Turbo (10KD, Sartorius, Göttingen, Germany) to obtain the end-product with a final volume of 500 μL. All the end-products from different mutant strains were loaded with the same volume of 20 μL to proceed sodium dodecyl sulfate-polyacrylamide gel electrophoresis (SDS-PAGE) and western blot in order to compare the expression level of total proteins and PA and LF, respectively. Grayscale analysis was performed using ImageJ v1.8.0 software (NIH, Bethesda, USA) for PA relatively quantification based on western blot images. Culture supernatants from candidate strains were administered to mice via the i.t. route to evaluate their safety. Experimental groups are listed in **Table S3**.

### Culture supernatant extract anthrax vaccine and its liquid and dry powder formulations preparation and characterization

Multiple batches of A16R-5.1 culture supernatant were prepared, then mixed thoroughly and named the CSE vaccine with minimized batch-to-batch variation. After being analyzed by SDS-PAGE and western blot, the CSE vaccine was dispensed and stored at – 80 °C.

Total protein concentrations of the CSE vaccine were measured by bicinchoninic acid assay (BCA, Gene-Star, Beijing, China). An enzyme-linked immunosorbent assay (ELISA, Dakewe, Beijing, China) was used to quantify the PA protein in the CSE vaccine according to a previously method [20]. Serially diluted commercial recombinant PA (rPA, 171E, List Labs, Campbell, CA, USA) was the reference standard. To identify the constituents of the CSE vaccine, the culture supernatant from starting strain A16R and the CSE vaccine were identified using tandem mass tags (TMT, A44522, Thermo Fisher Scientific, MA, USA) and liquid chromatography-tandem mass spectrometry (LC-MS/MS, EASY-nLC 1200 UPLC system, Thermo Fisher Scientific) as previously described [21]. The differentially expressed proteins (DEPs) were considered upregulated if |FC (A16R/CSE)| ≥ 1.2 or downregulated if |FC (A16R/CSE)| ≤ 0.83 with corrected *P*-values < 0.05. All spectra were searched against the *B. anthracis* genome in the GenBank database (https://www.ncbi.nlm.nih.gov/genbank/). The mass spectrometry data were analyzed with MaxQuant v. 2.1.4.0 (https://www.biochem.mpg.de/6304115/maxquant) [22]. Before immunization, the CSE vaccine was mixed into CpG oligonucleotide (CpG) adjuvant at a 1:1 ratio to form the CSE vaccine liquid formulations. The CSE dry powder formulation was prepared by the spray freeze drying (SFD) method as previously described [11]. The specific dry powder recipes are detailed in **Table S4**. The CSE dry powder was reconstituted in deionized water and analyzed by SDS-PAGE and western blot to determine the quality and stability. The immunogenicity of dry powder was checked by ELISA. Briefly, reconstituted dry powder and liquid vaccine were coated to plates (Corning, NY, USA) with the same quality of 10 μg, and plates were incubated with serum of three doses of immunized CSE vaccine, then their ability to combine IgG antibodies in the serum was examined and compared. Particle morphology was observed in multiple fields under a scanning electron microscope (S-3400N; Hitachi, Tokyo, Japan). The moisture content, volume median diameter (VMD), and mass median aerodynamic diameter (MMAD) of the dry powder vaccines were measured according to previously described methods [23].

### Animals and ethics statement

Female specific pathogen-free (SPF) B10.D2-*Hc^0^H2^d^H2-T18^c^*/oSnJ (hereafter, B10.D2-*Hc^0^*) mice aged 6–8 weeks were obtained from Jackson Laboratory (Bar Harbour, ME, USA) and maintained in the laboratory. This study was conducted with the permission of the Institute of Animal Care and Use Committee (IACUC) at the Beijing Institute of Microbiology and Epidemiology under Ethical Approval No. IACUC-IME-2021-033.

### Immunization

The B10.D2-*Hc^0^* mice were randomly divided into one of fourteen groups (44 per group) of which six were experimental groups, six were negative controls, and two were blank controls (**Table 1**). The mice were immunized by aerosolized intratracheal inoculation or subcutaneous injection (hereafter, i.t. and s.c., respectively) on days 0, 21, and 42 of the trial. The liquid and dry powder formulations were administered with MicroSprayer Aerosolizer or Dry Powder Insufflator (Huironghe Company, Beijing, China), respectively, following previously described methods [23].

**Table 1.** Summary of immunization groups used in the experiment.

Number	Immunization route	Group	Antigen	Adjuvant	Formulation type	Antigen dose (μg/mouse)	Adjuvant dose (μg/mouse)	Volume in total (μL/mouse)
1	i.t.	experimental	CSE	CpG	Powder	20	20	50
2			CSE	CpG	Powder reconstituted	20	20	50
3			CSE	CpG	Liquid	20	20	50
4		negative control	/	CpG	Powder	/	20	50
5			/	CpG	Powder reconstituted	/	20	50
6			/	CpG	Liquid	/	20	50
7		blank control	PBS	/	Liquid	/	/	50
8	s.c.	experimental	CSE	CpG	Powder reconstituted	20	20	100
9			CSE	CpG	Liquid	20	20	100
10			CSE	Alum	Liquid	20	100	100
11		negative control	/	CpG	Powder reconstituted	/	20	100
12			/	CpG	Liquid	/	20	100
13			/	Alum	Liquid	/	100	100
14		blank control	PBS	/	Liquid	/	/	100

### rPA-specific antibody level determination by ELISA

Sera and bronchoalveolar lavage fluids (BALFs) were collected from mice (three per group) after each immunization. The antigen-specific immunoglobulin G (IgG), IgG1, and IgG2a levels in the sera and the IgG and secretory immunoglobulin A (SIgA) levels in the BALFs were determined by ELISA according to previously described methods [11]. All measurements were performed at least in triplicate.

### Toxin-neutralizing antibody (TNA) assay

To evaluate the neutralizing activity of the sera from the vaccinated mice, a cytotoxicity analysis based on mouse macrophage J774A.1 cells was performed according to a previously described protocol [11]. Briefly, J774A.1 cells (5 × 10^4^/well, Procell, Wuhan, China) were incubated overnight. LeTx was added to the cells along with a serum dilution sample and the cells were enumerated with a CCK-8 kit (Dojindo Laboratories, Kumamoto, Japan) and incubated for 4 h. The OD_450_ was measured in a SpectraMax i3x (Molecular Devices, CA, USA). The four-parameter logistic sigmoid regression curve was used to determine the dilution of the antiserum, which reduced the toxicity of LeTx by 50%. All measurements were performed at least in triplicate.

### Determination of T cell cytokine secretion

After the third inoculation, mice (three per group) were euthanized and their spleens were excised to prepare single-cell suspensions. The interferon-γ (IFN-γ) and interleukin-4 (IL-4) levels in the samples were determined by enzyme-linked immunospot (ELISPOT) assay following previously described [24]. All measurements were performed at least in triplicate.

### Lymphocyte proliferation assay

A lymphocyte proliferation assay was conducted according to the revised version of a previously method [25, 26]. Single-cell suspensions (4 × 10^5^/well) were seeded in plates containing RPMI 1640 medium containing 10% (v/v) fetal bovine serum, 1% (v/v) penicillin–streptomycin (Thermo Fisher Scientific), and 20 µg/mL rPA at 37 °C and under 5% CO_2_ for 72 h. During the final four hours of incubation, 20 µL CCK-8 solution was added. All measurements were performed at least in triplicate.

### Bacillus anthracis aerosol challenge

A *B. anthracis* aerosol challenge was conducted on day 63 post-primary inoculation (dppi) via the i.t. route. Challenge doses of 100 × LD_50_ and 200 × LD_50_ *B. anthracis* spores were targeted using LD_50_ = 2.5 × 10^3^ spores/animal [11]. Morbidity and mortality were observed and recorded daily for 14 d.

On days 2, 14, and 28 after the *B. anthracis* aerosol challenge, three mice per group were sacrificed and their lungs, spleens, livers, and blood were harvested. Tissue homogenates and whole blood were serially diluted and coated onto tryptic soy agar (TSA, BD Biosciences) plates. The bacterial colonies were enumerated and their corresponding concentrations were calculated and expressed in colony-forming units (CFU)/g or CFU/mL.

### Histopathology

The lung, spleen, and liver tissues were collected from mice (three per group) at necropsy at 63 dppi and two days post-challenge. The tissues were soaked in 4% (v/v) paraformaldehyde (PFA), embedded in paraffin, sectioned, and stained with hematoxylin and eosin (H&E). Microscopic lesion severity was graded using a five-level system, namely, no pathological damage, minimal, mild, moderate, or severe. A trained pathologist double blinded to the treatments performed these assessments.

### Comparative immunogenicity and protective effects of CSE and rPA liquid formulations

B10.D2-*Hc^0^* mice (15 per group) were immunized via the i.t. route to compare the immunogenicity and protective effects of CSE and rPA liquid formulations. The mice were randomly assigned to four groups as listed in **Table S5**. ELISA was used to measure the serum and BALF antigen-specific IgG levels after each immunization and every third immunization, respectively. At 63 dppi, 2 × 10^6^ *B. anthracis* spores were delivered to the B10.D2-*Hc^0^* mice via the i.t. route. Morbidity and mortality were observed and recorded daily for 14 d.

### Statistics

All data were presented as means ± standard deviation (SD). All statistical analyses were performed using GraphPad Prism v. 8.0.2 (La Jolla, CA, USA). The antibody levels and bacterial loads were determined by two-way analysis of variance (ANOVA) followed by the least significant difference (LSD) or Tukey's test. The IFN-γ and IL-4 levels were compared by one-way mutation analysis followed by the LSD test. Survival rates were analyzed using Kaplan-Meier survival estimates. The relative expression level of PA in the mutant strains was compared by one-way ANOVA, followed by Tukey's test. The relative expression level of PA in the A16R-5 and A16R-5.1 strains were compared by the F test. Differences between treatment means were considered significant at *P* < 0.05.

## Results

### Construction and characterization of B. anthracis mutants deficient in extracellular protease activity

To obtain the high immunogenicity and safety of the CES anthrax vaccine, we knocked out six protease activity-associated genes in sequence so as to reduce the degradation of the PA antigen and avoid the potential toxicity caused by proteases. The resultant strain derivatives were designated *B. anthracis* A16R-1 to – 6 (**Table S1**). PCR identification showed that the discrepancy in amplified fragment length between every two adjacent mutant strains aligned with the size of the knocked-out target gene. Hence, the serial target genes were all successfully knocked out (Figure 1B). From the results of western blot and ImageJ analysis **(**Figure 1C, D), we can see that in the supernatants of equal volume (20 μL), the express levels of PA were maintained at very low levels firstly in the strains A16R-1 and A16R-2, and then increased step-by-step in A16R-3, A16R-4, and A16R-5, but dropped off again in A16R-6 (*P* < 0.05).

Therefore, the A16R-5 strain with the highest PA expression level was selected for the next safety evaluation. Unfortunately, immunization with A16R-5 culture supernatants via the i.t. route resulted in mice mortality (Figure 1G). In order to further remove bacterial virulence, the *lef* gene encoding LF (which can bind to PA to form lethal toxin that can target cellular pathways, thereby inhibiting host responses and causing lethality [2]), was deleted from A16R-5 to create strain A16R-5.1. Western blot and ImageJ results showed that the relative expression levels of PA in the A16R-5 and A16R-5.1 strains were comparable (*P* > 0.05, Figure 1E and F),while LF was completely lost in A16R-5.1 (Figure 1E). Lastly, A16R-5.1 culture supernatants were administered to mice via the i.t. route, and all the mice survived; none showed any clinical symptoms, even those in the high-dose (50 µg per mouse) group (Figure 1G). Hence, the attenuated mutant strain A16R-5.1 with lower protease activity and virulence was identified as the candidate for preparing a CSE anthrax vaccine.

### Characterization of CSE anthrax vaccine

The total protein and PA concentration in the CSE vaccine were measured by BCA and ELISA respectively, whose standard curves with R^2^ = 0.99 are shown in Figures S1 and S2. It was determined that the concentration of total protein and PA was 0.97 and 0.48 mg/mL respectively. Thus, the PA accounted for 49.5% of the total protein.

In addition, LC-MS/MS was used to compare the composition differences between CSE and the original strain. The results of differential protein analysis showed that there were 558 significantly upregulated and 217 significantly downregulated proteins in the deletion mutant compared to the A16R strain (Figure 1H). The top 20 most abundant proteins were identified by an intensity-based-absolute-protein-quantification (iBAQ) algorithm (**Table S6**).

### Characterization of dry powder CSE anthrax vaccine

**Figure 2.**
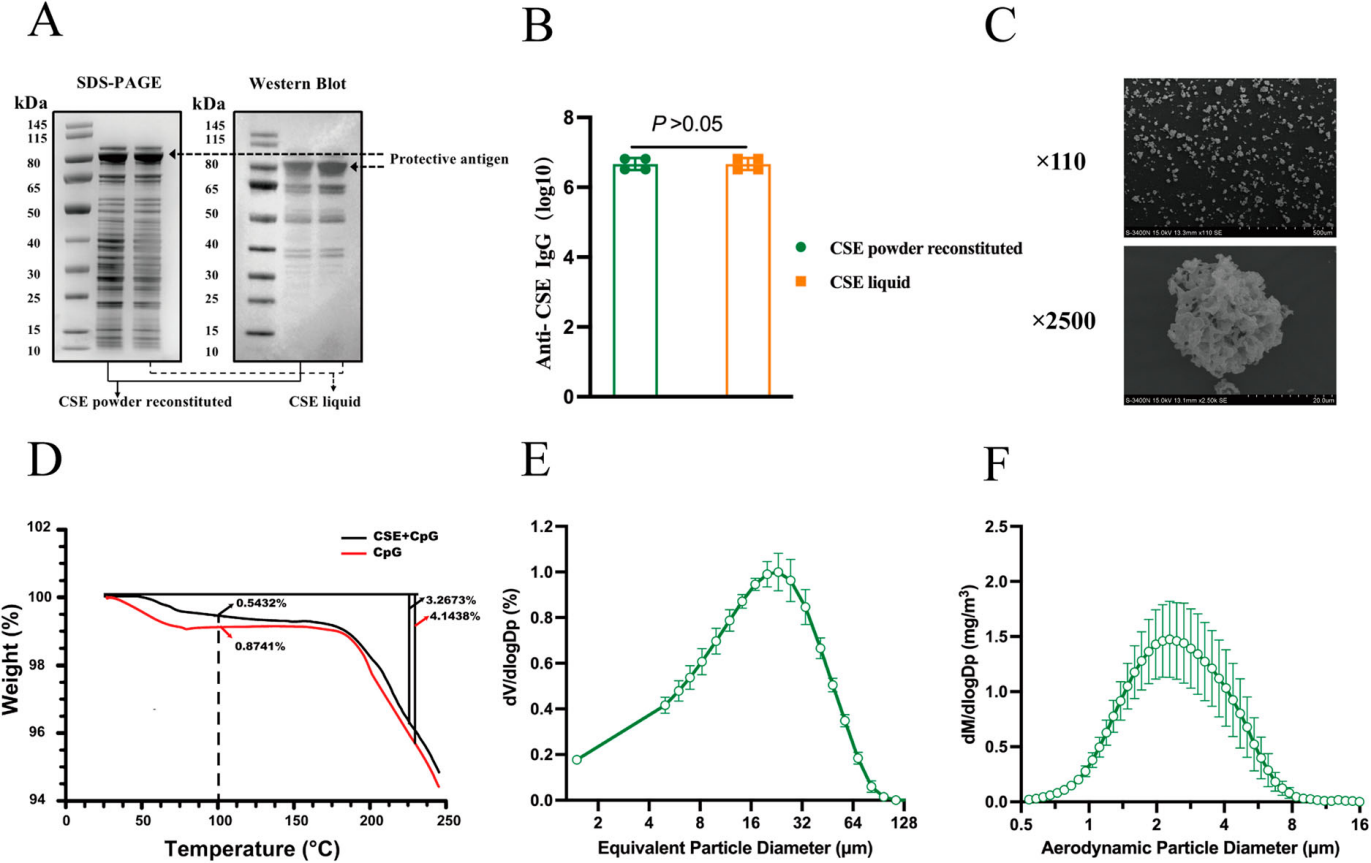
Characterization of CSE vaccine dry powder. **(A)** CSE dry powder and liquid analyzed by SDS-PAGE and western blot analysis with mouse anti-CSE polyclonal antibodies. **(B)** Immunogenicity of CSE dry powder and CSE liquid analyzed by ELISA with mice immunized with three CSE vaccines. **(C)** Scanning electron microscopy (SEM) images of CSE dry powder. **(D)** Moisture content of CSE and CpG dry powders (control). **(E)** VMD and **(F)** MMAD of aerosolized CSE dry powder.

In order to evaluate the suitability for inhalational immunization, the CSE vaccine dry powder particles were characterized in detail. The protein expression levels in the CSE dry powder reconstituted in PBS resembled those in the CSE liquid (Figure 2A), and the titers of IgG antibodies combined by the two formulations with equal quality were also comparable (*P* > 0.05, Figure 2B). Therefore, the protein integrity and immunogenicity of the dry powder vaccine prepared by SFD technology were not altered compared to the liquid vaccine. The SFD powder particles were porous and nearly spherical (Figure 2C) and their residual water content was ∼0.5432% (w/w) (Figure 2D). The VMD and MMAD of the dry vaccine powder were 19.42 ± 0.98 μm (Figure 2E) and 2.69 ± 0.28 μm (Figure 2F), respectively. The foregoing results indicated that dry powder vaccine prepared by SFD technology is suitable for aerosol inhalation [27].

### Humoral immune responses of mice immunized with CSE vaccines

To evaluate the effect of the prepared CSE vaccine on inducing humoral, lung mucosal, and cellular immunity and neutralizing antibodies in mice, we immunized mice with three CSE vaccine formulations (liquid, powder and powder reconstituted) and different adjuvants (CpG and Alum) by different routes (i.t and s.c) at 0, 21 and 42 dppi, respectively.

**Figure 3.**
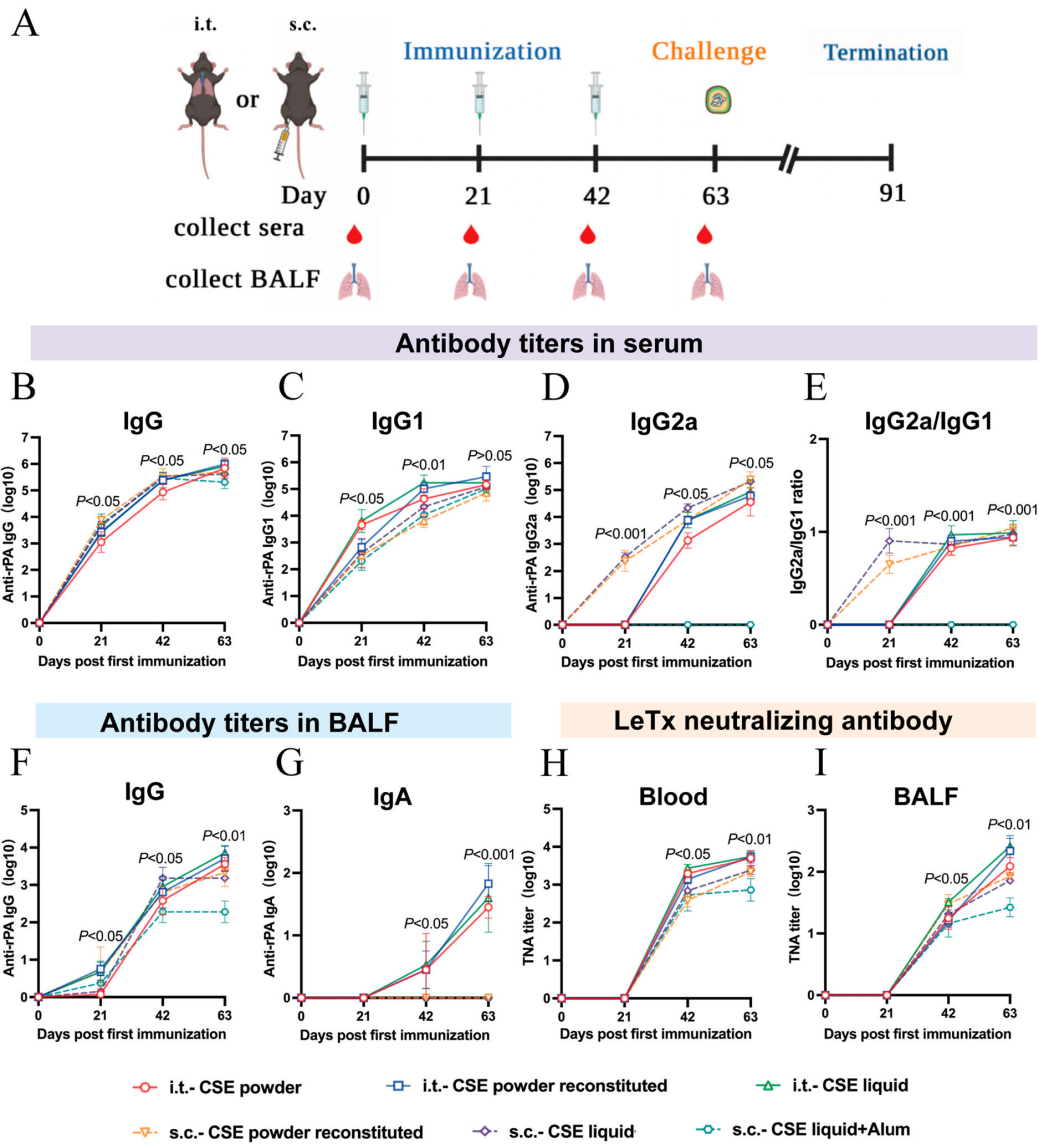
Immune responses and LeTx neutralizing antibodies after mice were immunized with different CSE vaccines. **(A)** Schematic timeline of immunization, challenge, and sera and BALF collection. **(B, C, D, E)** Reciprocal titers of **(B)** IgG, **(C)** IgG1, **(D)** IgG2a, and **(E)** IgG2a/IgG1 antibodies to CSE in serum. **(F, G)** Reciprocal titers of **(F)** IgG and **(G)** IgA antibodies to CSE in BALF. **(H, I)** Neutralizing antibody titers to CSE in **(H)** serum and **(I)** BALF. TNA titers were determined as inflection points on antibody dilution curves and reported as effective dilution at 50% inhibition of anthrax LeTx toxicity. Values were then log10-transformed.

For humoral immunity evaluation, antigen-specific IgG, IgG1, and IgG2a titers were determined by ELISA at 0, 21, 42, and 63 dppi (Figure 3A). We observed progressively higher levels of anti-rPA antibodies IgG, IgG1, IgG2a in all experimental groups, except for the alum adjuvant group which did not cause changes in IgG2a antibody titers (Figure 3B–D). At 63 dppi, serum-specific IgG levels of three i.t. route groups were comparable to those of two s.c. route groups with the CpG adjuvant (*P* > 0.05, Figure 3B), but were obviously higher than those of s.c. route group with the alum adjuvant (*P* < 0.05, Figure 3B). The IgG2a/IgG1 ratio of the s.c.-CSE liquid + alum group was almost 0, but those of all other experimental groups approached 1 (Figure 3E).

The preceding outcomes demonstrated that: (i) adjuvant type can have a significant effect on humoral immunity, with the CpG immunization being more effective than the alum adjuvant; (ii) the Th1/Th2 reaction induced by the CpG adjuvant was balanced, whereas the alum adjuvant tended to induce Th2 immune response; and (iii) in the case of the same adjuvant, the humoral immunity induced by different routes and formulations were comparable.

### Lung mucosal immune responses in mice immunized with CSE vaccines

For lung mucosal immunity evaluation, antigen-specific IgG and SIgA levels in BALFs were determined by ELISA at 0, 21, 42, and 63 dppi (Figure 3A). At 42 dppi, booster immunization significantly upregulated the anti-rPA IgG antibodies in all experimental groups. At 63 dppi, the various vaccines with CpG adjuvant induced comparable anti-rPA IgG titers regardless of routes or formulations, which were significantly higher than induced by the vaccine with the alum adjuvant (*P* < 0.01, Figure 3F).

As predicted, no specific SIgA was detected in the BALFs of the s.c.-immunized mice. On the contrary, in the three i.t.-immunized mice groups, anti-rPA SIgA appeared firstly at 42 dppi and enhanced rapidly after three doses. At 63 dppi, there were no significant differences among the three formulations via the i.t. route (liquid, dry powder, powder reconstituted) (*P* > 0.05, Figure 3G).

The results demonstrated that (i) as with humoral immunity, adjuvant type affects the effect of immunization; (ii) the activation of mucosal immunity is mainly influenced by the route of vaccination; (iii) the i.t. route can effectively activate mucosal immunity marked by SIgA.

### Toxin neutralization assay of mice immunized with CSE vaccines

To determine the effect of CSE vaccines in neutralizing toxins *in vitro*, sera and BALFs were collected to assess functional antibody development at 0, 21, 42, and 63 dppi (Figure 3A). At 63 dppi, the serum neutralizing antibody titers in mice immunized via the i.t. route were not significantly different from those in mice immunized via the s.c. route with the CpG adjuvant (*P* > 0.05, Figure 3H), but were significantly higher than those in mice immunized via the s.c. route with the alum adjuvant (*P* < 0.05, Figure 3H). The neutralizing antibody titers of the BALFs from the mice immunized with i.t.-liquid and i.t.-powder reconstituted were significantly higher than those of the BALFs from the mice immunized with i.t.-powder, s.c.-powder reconstituted, and s.c.-liquid. Nevertheless, mice immunized with s.c.-liquid adding alum had the lowest neutralizing antibody titers in BALFs (*P* < 0.05, Figure 3I).

Overall, (i) for adjuvants, the alum adjuvant had the lower activation capacity for neutralizing antibodies in both the blood and lungs than the CpG adjuvant; (ii) regarding the different routes and formulations, their activation capacities for neutralizing antibodies were comparable in the blood, but were different in the lungs; and (iii) the liquid formulation immunized via the i.t. route better induced neutralizing antibodies in the lungs.

### Cellular immune responses of mice immunized with CSE vaccines

**Figure 4.**
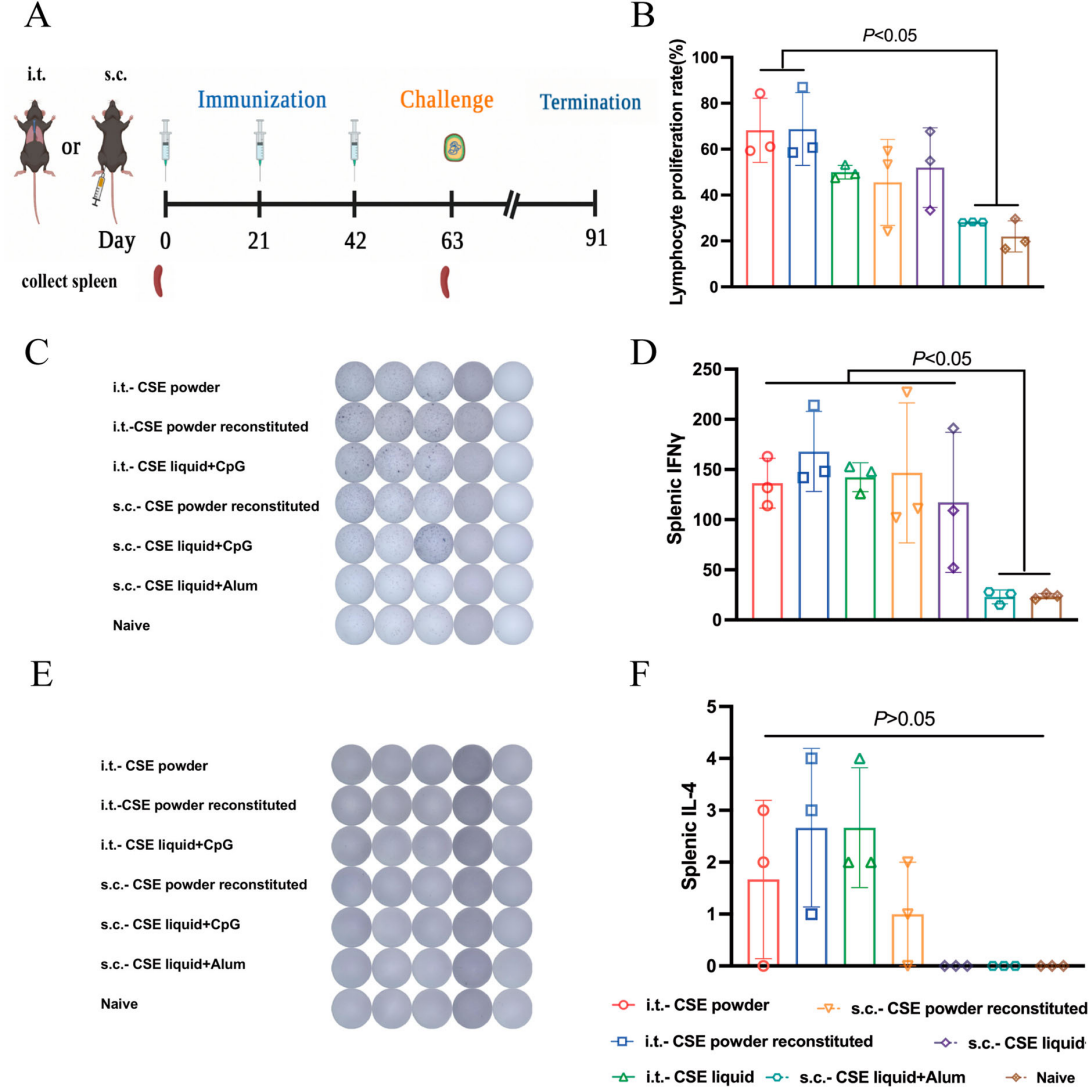
Antigen-specific T cell immune and lymphocyte proliferation responses in mice immunized with different CSE vaccines. **(A)** Schematic timeline of immunization, challenge, and spleen collection. **(B)** T lymphocyte proliferation assay. **(C, E)** ELISPOT and antigen-specific Biosys Bioreader 7000 determinations of **(C)** IFN-γ and **(E)** IL-4. **(D, F)** Antigen-specific measurements of **(D)** IFN-γ and **(F)** IL-4.

For cellular immunity evaluation, we then observed splenic lymphocyte proliferation in each treatment group at 0, 21, 42, and 63 dppi (Figure 4A). The lymphocyte proliferation rates in the mice immunized with i.t.-powder and i.t.-powder reconstituted were significantly higher than those in the control mice and in the mice immunized with s.c.-liquid adding alum (*P* < 0.05, Figure 4B). The lymphocyte proliferation rates did not significantly differ among the remaining groups.

The IFN-γ secretion levels did not significantly differ among the CpG adjuvant immunization groups but were significantly higher for the s.c.-liquid adding alum and control groups than the others (*P* < 0.05, Figure 4C and D), indicating that CpG adjuvant immunization more effectively induced the cellular immune response than alum adjuvant immunization. However, there were no significant differences among the CSE vaccines in terms of the IL-4 secretion levels they induced (*P* > 0.05, Figure 4E and F).

### Protection of mice against i.t. challenge with B. anthracis spores

**Figure 5.**
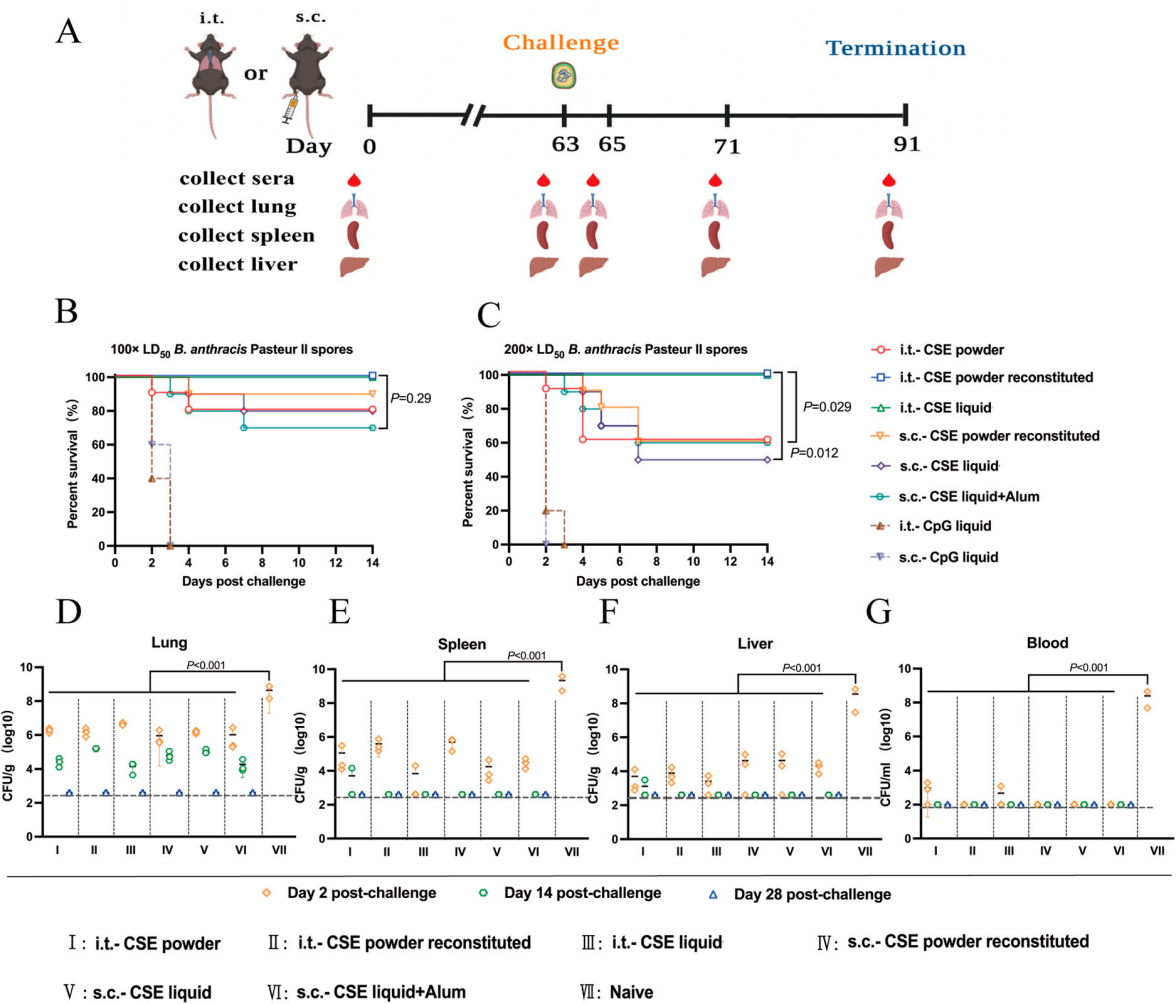
Protective efficacies of CSE vaccines against aerosolized *B. anthracis* spore challenges. **(A)** Schematic timeline of aerosol *B. anthracis* spore challenge and organs collection. **(B, C)** Survival of mice (n = 10) following **(B)** 100 × LD_50_, **(C)** 200 × LD_50_ aerosolized *B. anthracis* spore challenge, respectively. **(D – G)** Bacterial and spore loads of mice euthanized at days 2, 14, and 28 post 100 × LD_50_ aerosolized *B. anthracis* spore challenge. **(D)** lungs, **(E)** spleens, **(F)** livers, and **(G)** blood. Limits of detection (LoD) were 400 CFU for lungs, spleens, and livers and 100 CFU for blood. Challenges with 100 × LD_50_ *B. anthracis* spores were performed twice on different lots of immunized mice.

To determine the immunoprotective effect of various CSE vaccines, we then assessed the efficacy with which vaccines protected the mice against i.t. challenge with 100 × LD_50_ or 200 × LD_50_ *B. anthracis* spores (Figure 5A). The control group comprised six negative controls and two blank controls. Individuals from all the control groups therein died within 3 d following the spore challenge. For simplicity, only the i.t.-CpG and s.c.-CpG liquid subgroups are shown. When the challenge dose was 100 × LD_50_, even though only i.t.-liquid and i.t.-powder reconstituted groups survived in whole, there were no significant differences among the experimental groups (*P* > 0.05, Figure 5B). We then increased the challenge dose to 200 × LD_50_, and the i.t.-liquid and i.t.-powder reconstituted groups still achieved 100% protection efficiency, which was much higher than the protection efficiency equal to or even lower than 60% caused by the other experimental groups (*P* < 0.05, Figure 5C). Unsurprisingly, the results of 200 × LD_50_ challenge experiment were consistent with the results of the toxin neutralization assay.

The bacterial and spore loads in the lungs, spleens, livers, and blood of the mice challenged with 100 × LD_50_ via the i.t. route are shown in Figure 5D–G. All mice in the control groups were uniformly denoted as naïve, bacterial load data were only available within 2 d of the challenge. At day 2 post-challenge, in the lungs, spleens, livers and blood of the control mice, the bacterial and spore counts were significantly higher than those of the mice in the immunization groups, respectively (*P* < 0.001). In the latter, at minimal dilution, no bacteria or spores were detected in the spleen, liver, and blood and the lungs of all mice until 14 and 28 days post-challenge, respectively.

In summary, as with the toxin neutralization assay results, liquid formulations administered via the i.t. route were resistant to higher doses (200 × LD_50_) of inhalation challenge, which appeared to be the most optimal inoculation strategy.

### Histopathological analyses

**Figure 6.**
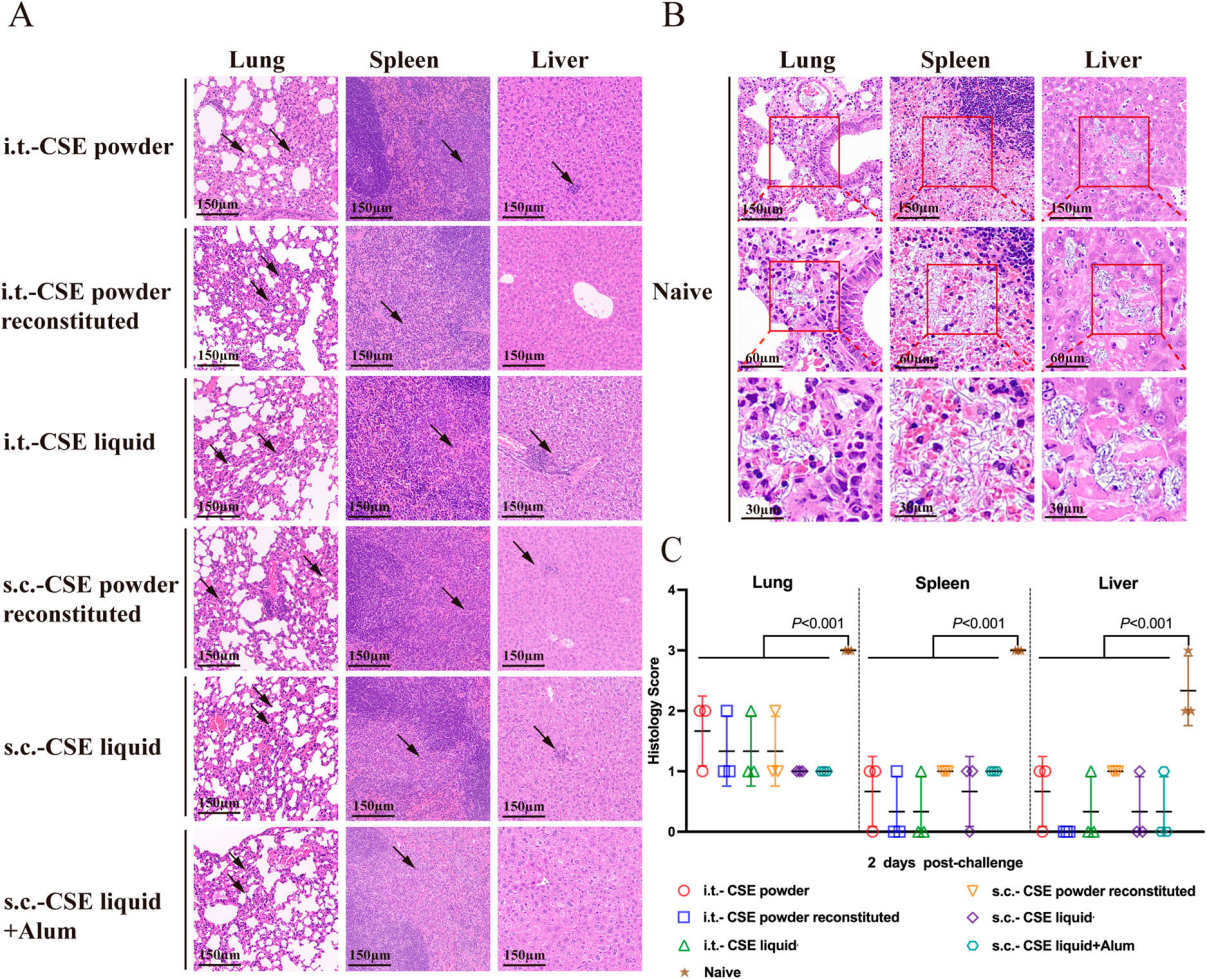
Pathological changes in tissues of CSE vaccine-immunized mice after aerosolized *B. anthracis* spore challenge. Mice (three per group) were euthanized day 2 post 100 × LD_50_ i.t. *B. anthracis* spore challenge. Portions of lungs, spleens, and livers were collected. **(A)** H&E staining of tissues from vaccinated mice. Black arrows indicate neutrophil or lymphocyte infiltration. **(B)** H&E staining of tissues of control mice. Red square indicates enlargement. **(C)** Tissue sections were evaluated as follows: 0, no pathological lesions; 1, minimal; 2, mild; 3, moderate; 4, severe.

To verify the safety and immunoprotective effect of the CSE vaccine, the organs were observed for pathological changes. No significant pathological changes were detected in the lungs, spleens, or livers after three immunizations in all groups (data not shown). Hence, CSE administered by the i.t. and s.c. routes was safe. When the challenge dose was 100 × LD_50_, we investigated pathological tissues in these organs of mice administered 100 × LD_50_ spores after 2 days. In the vaccinated mice, all the foregoing organs presented with slight inflammatory cell infiltration (Figure 6A). In contrast, bacilli were clearly visible in the same organs of the control mice (Figure 6B). Thus, the pathological scores were significantly higher for the control mice than the vaccine-immunized mice (*P* < 0.001, Figure 6C).

### Comparative immunogenicity and efficacy of CSE and rPA liquid formulations

**Figure 7.**
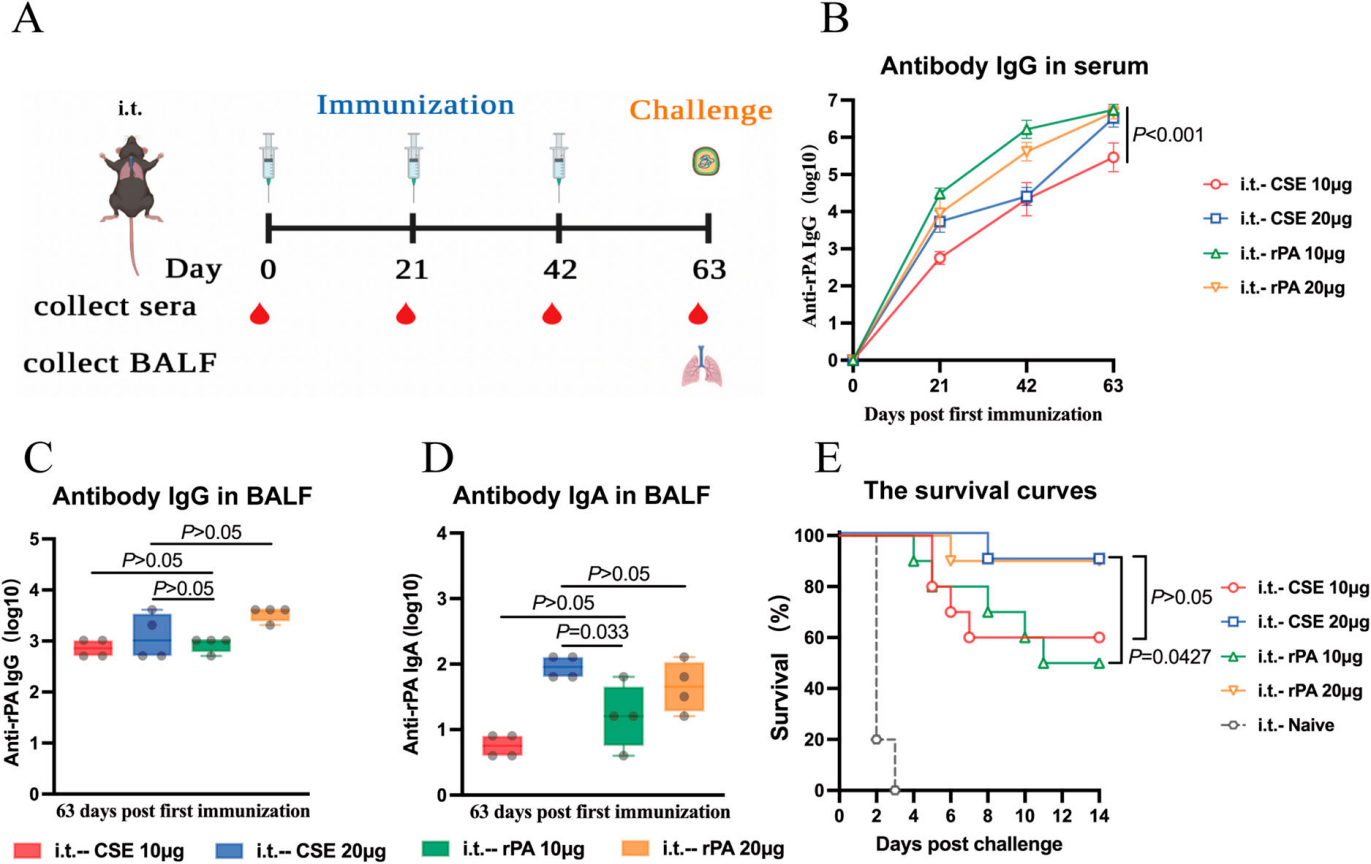
Immune responses and relative protective efficacies of CSE vaccine and rPA. **(A)** Schematic timeline of immunization and challenge. **(B)** Reciprocal IgG titers in serum. **(C, D)** Reciprocal **(C)** IgG and **(D)** IgA titers in BALF. **(E)** Survival of mice (n = 10) against 100 × LD_50_ aerosolized *B. anthracis* spore challenge.

We then compared the immunogenicity and protective efficacy of various CSE and rPA liquid formulation doses (Figure 7A). At 63 dppi, the serum IgG antibody titers did not significantly differ among the i.t.−20 µg CSE (including 10 µg PA), i.t.−20 µg rPA, and i.t.−10 µg rPA treatment groups. However, they were all significantly higher than that of the i.t.−10 μg CSE (including 5 µg PA) treatment group (*P* < 0.001, Figure 7B). In the BALFs, there were no significant differences in the IgG antibody titers at the same dose of total protein (i.t.−20 µg CSE and i.t.−20 µg rPA; i.t.−10 µg CSE and i.t.−10 µg rPA) or PA (i.t.−20 µg CSE and i.t.−10 µg rPA) (*P* > 0.05, Figure 7C). Conversely, the CSE vaccine induced higher titers of SIgA than rPA at the same PA content (*P* < 0.05, Figure 7D).

When the qualities of the total protein were the same, there was no significant difference in survival rate between immunized groups. Interestingly, when the qualities of PA were the same, the survival rate of i.t.−20 μg CSE was significantly higher than that of i.t.−10 µg rPA (*P* = 0.0427, Figure 7E). This indicated that the protection of the CSE vaccine with complex components was superior to that of using rPA alone.

## Discussion

*B. anthracis* is the causative agent of pulmonary anthrax which is the most aggressive and lethal form of this disease. This pathogen has been intentionally deployed as a bioweapon and can transmit anthrax as an aerosol to a large human or other mammalian population [28]. In this study, we constructed *B. anthracis* A16R-5.1 strain with low protease activity and utilized it to prepared various CSE vaccine formulations in the attempt to prevent pulmonary anthrax. We evaluated the potency and efficacy of the CSE anthrax vaccine against a lethal aerosol challenge of *B. anthracis* spores and found that (i) the i.t. route (powder reconstituted and liquid formulations) conferred significantly stronger immunoprotection than the s.c. route; (ii) the powder reconstituted and liquid formulations elicited similar immunoprotection by the same route of administration, both being superior to the dry powder formulation; and (iii) the CpG adjuvant was significantly more effective than the alum adjuvant.

AVA has provided good protection against pulmonary anthrax. As it contains the LF component, however, it may cause adverse reactions if frequently injected [29]. Therefore, further attenuation of the AVA is necessary. As the starting strain (V770-NP1-R) of AVA is now not available in our country, A16R, approved in China, being of the same capsule-deficient phenotype as V770-NP1-R [6, 15], was selected as the original strain in this study. However, V770-NP1-R strain has lower protease activity, resulting in limited degradation of the secreted protective antigen. Herein, we decided to construct a mutant strain with low protease activity based on A16R for the preparation of CSE vaccines, and then a strategy of sequential knockout of six protease genes was adopted. Our results showed that up to A16R-5, the more protease genes were knocked out, the higher the PA level. Unexpectedly, in A16R-6 (A16R-5*Δ htrA*), PA levels instead decreased. However, one previous study showed that deleting *htrA* did not appear to affect the level of PA produced in the Sterne strain [30]. Since HtrA can play a regulatory role and affect the expression of more than 1000 genes [31], and its effect on secretory proteins is obviously different in various media conditions [32], it is speculated that knockout of *htrA* plus other protease gene or genes may cause complex regulatory mechanisms such as compensatory protein degradation under the culture condition of our study. Nevertheless, the detailed reason for this phenomenon remains to be explored. Eventually, compared to AVA, the CSE vaccine prepared here was found to: (i) knock out five protease activity-associated genes and the *lef* gene to reduce PA degradation and enhance immunoprotection; (ii) knock out the LF encoded gene, thereby avoiding lethal toxin formation and improving vaccine safety; and (iii) has superior inoculation efficiency, as it is administered by the i.t. route.

In our previous study on rPA [11], 20 × LD_50_ inhaled spores were administered to mice. Here, however, this dosage was increased to 100 × LD_50_, the CSE vaccine still conferred full protection after it was administered via the i.t. route. Relative to the rPA, the multicomponent CSE vaccine provided enhanced immunoprotection against pulmonary anthrax when both vaccines have the same PA content. We postulate that other components of the CSE vaccine besides PA might have immunoprotective efficacy similar to that of PA. In a multiepitope chimeric vaccine study, equal doses of ID-LFn and PA exerted the same immunoprotective effect [33]. PA-based anthrax vaccines have comparatively less efficacy against the toxin than live attenuated Sterne vaccines. Thus, other antigens present may provide additional protective immunity [34]. Other studies also showed that complex antigen compositions may have a stronger immunostimulatory effect than single antigen. Hence, the latter may not suffice against inhalation infection challenges [35, 36]. The foregoing findings corroborated our hypotheses.

The aerosolized i.t. inoculation used herein ensures accurate drug dosing [37] and uniform pulmonary distribution and deposition [38]. Hence, it is a preferable method of pulmonary vaccine delivery utilized in surrogate animal model. Pulmonary vaccine delivery has several advantages over traditional injection vaccination. It readily releases antigens and requires fewer antigens than the s.c. route to provide the same level of immunoprotection [39]. Moreover, it is able to trigger mucosal antibody SIgA, thereby preventing the penetration of infectious agents, and confers rapid protection [40]. From this work, we have determined that the i.t. route significantly promotes SIgA antibody secretion whereas the s.c. route does not. The immunoprotective effects of i.t. administration (powder reconstituted and liquid formulation) are significantly better than those of s.c. administration. Similar results were reported in a study comparing the relative efficacies of influenza vaccines administered via the intratracheal and intramuscular routes [41]. In addition, needle-free vaccination avoids the adverse effects caused by injections, reduces the risks associated with needle-stick injuries, requires less assistance and intervention by health care professionals, and improves patient compliance [42]. Thus, pulmonary delivery vaccination has become increasingly popular. For instance, aerosolized adenovirus type-5 vector-based COVID-19 vaccine (Ad5-nCoV) has been in high demand in recent years [43]. The aerosolized i.t. inoculation was conducted by rodent-specific devices in this study. Similarly, there are many mature inhaler devices for human pulmonary vaccination, such as pressurized metered-dose (pMDI), soft mist (SMI), smart inhalers, and dry powder inhalers (DPIs) [44].

Dry powder formulations have superior storage stability and require no cold chain transport. Thus, their distribution and coverage can be greater than those of perishable liquid formations [45]. Unfortunately, the dry powder formulation made in this study presented inadequate protection against high dose (200 × LD_50_) aerosol challenge with *B. anthracis* spores. However, interestingly, when the dry powder was reconstituted into liquid, it would regain full protection. The underlying mechanism of this phenomenon needs to be explored; meanwhile, we suggest that the CSE vaccine could be distributed and stored as a dry powder and inoculated as a redissolved liquid formulation. A similar strategy was used to prepare a recombinant dry powder VLP (Virus-Like Particle) vaccine against human papillomavirus [46].

In the present study, we demonstrated that the CSE anthrax vaccine administered intratracheally induces strong humoral, cellular, and mucosal immunity in mice, confers complete protection against aerosolized *B. anthracis* spore challenges at much higher doses than those used in previous studies, and had greater immunoprotective efficacy than the rPA vaccine. Taken together, we believe that our study makes a significant contribution to the literature because it empirically demonstrated that the CES anthrax vaccine developed herein is suitable and scalable for use in inhalational immunization against fatal pulmonary anthrax. In future work, the components of the CSE vaccine and its mechanism subsequently need to be investigated in depth. More importantly, if the opportunity arises, we aim to consider the construction of a more optimized mutant strain for vaccine preparation and comparison with the AVA vaccine in follow-up work.

## Supplementary Material

Supplemental Material
