# Supplementary material for: Inhalable vaccine of bacterial culture supernatant extract mediates protection against fatal pulmonary anthrax
Source: Emerg Microbes Infect. 2023 Mar 31;12(1):2191741. doi: 10.1080/22221751.2023.2191741 (PMC10071900; doi:10.1080/22221751.2023.2191741)
Supplement: Supplemental Material [file TEMI_A_2191741_SM2169.docx]

**Supplemental material**

Inhalable vaccine of bacterial culture supernatant extract mediates protection against fatal pulmonary anthrax

Li-Na Zhai^1,2^**^†^**, Yue Zhao^2,4†^, Xiao-Lin Song^2^, Tong-Tong Qin^2^, Zhi-Jun Zhang^2^, Jia-Zhen Wang^2^, Cheng-Yu Sui^2^, Li-Li Zhang^2^, Meng Lv^2^, Ling- Fei Hu^2^, Dong-Sheng Zhou^2^, Tong-Yu Fang^2^, Wen-Hui Yang^2^*, Yan-Chun Wang ^3^*

Supplementary Tables

**Table S1.** The bacterial strains, plasmids used in this study.

| Strains /Plasmids | | Relevant characteristics | Source |
| --- | --- | --- | --- |
| *B．anthracis*  Strains | A16R | pXO1^+^ pXO2^－^, China vaccine strain, host for genome editing | This lab |
|  | A16R-1 | *B.anthracis* A16R with *nprR* gene deletion | This study |
|  | A16R-2 | *B.anthracis*A16R-1 with *mmzp*gene deletion | This study |
|  | A16R-3 | *B.anthracis*A16R-2 with *inhA1*gene deletion | This study |
|  | A16R-4 | *B.anthracis*A16R-3 with *tasA*gene deletion | This study |
|  | A16R-5 | *B.anthracis*A16R-4 with *GBAA_2860*gene deletion | This study |
|  | A16R-5.1 | *B.anthracis*A16R-5 with *lef*gene deletion | This study |
|  | A16R-6 | *B.anthracis*A16R-5 with *GBAA_3660*gene deletion | This study |
|  | Pasteur II | pXO1+ pXO2+, veterinary anthrax vaccine | This lab |
| *Escherichia coli*  Strains | Top 10 | *F-lacX74 recA1 araD139 Δ(mcrA Δ(mrr-hsd RMS-mcrBC)80lacZΔ M15Δ(ara-leu) 7697 galU galK rpsL (StrR) endA1 nupG* | TianGen, China |
|  | SCS110 | *rpsL (*Strr*) thr leu endA thi-1 lacY galK galT ara tonA tsx damdcm supE44 Δ(lac-proAB) (F´ traD36 proAB lacIq ZΔM15)* | Transgen, China |
| Plasmids | pJOE8999 | Rep pE194 (Ts), Kanr, Pman-cas9, PvanP*-sgRNA9, shuttle vector | [1] |
|  | pJOE*-nprR* | pJOE8999 with Cas9 and homologous arms of *nprR* from B. anthracis A16R | This study |
|  | pJOE*-inhA1* | pJOE8999 with Cas9 and homologous arms of  *inhA1* from B. anthracis A16R | This study |
|  | pJOE*-tasA* | pJOE8999 with Cas9 and homologous arms of  *tasA* from B. anthracis A16R | This study |
|  | pJOE*-mmzp* | pJOE8999 with Cas9 and homologous arms of *mmzp*from B. anthracis A16R | This study |
|  | pJOE*-GBAA_2860* | pJOE8999 with Cas9 and homologous arms of *GBAA_2860* from B. anthracis A16R | This study |
|  | pJOE*-lef* | pJOE8999 with Cas9 and homologous arms of  *lef*  from B. anthracis A16R | This study |
|  | pJOE*-GBAA_3660* | pJOE8999 with Cas9 and homologous arms of *GBAA_3660* from B. anthracis A16R | This study |

**Table S2.** List of primers and their sequences used for construction of anthrax protease deletion mutant strains.

| Primer | sgRNA  Sequence (5’-  3’) | Identification of Sequence (5’-  3’) |
| --- | --- | --- |
| *nprR*-F | tacgACACAAGAAGAATTATGTCA | CAGCGCGTGTGCCCCAAAGGG |
| *nprR*-R | aaacTGACATAATTCTTCTTGTGT | TTGACCAACAATATCAGGTTTACTG |
| *inhA1*-F | tacgAAGTTAACACGAATCATACC | TTTGGAGACACCAGAGTTCATTG |
| *inhA1*-R | aaacGGTATGATTCGTGTTAACTT | GTTCTAACGTAAGCGGCGTCAGCTG |
| *tasA*-F | tacgTCTTTTTCAACTGTATCACC | TAGTGCTACGCCGAAATACAAAAG |
| *tasA*-R | aaacGGTGATACAGTTGAAAAAGA | GCAAGACACGAAAAGAAGGTGAGTG |
| *mmzp*-F | tacgATAGGAATAGGTCACAACAG | TTATCTTTGATGGTTGAATCTATG |
| *mmzp*-R | aaacCTGTTGTGACCTATTCCTAT | AGTGGGGTAGGTTAAGTTGATTTTG |
| *GBAA_2860*-F | tacgCAAGTTGAAGGTATGTCTTG | GTTCCGAAGAACCGATAGATTGAATG |
| GBAA_2860-R | aaacCAAGACATACCTTCAACTTG | GGTAACTGTTGAAGGAACTTCAGTAG |
| *lef*-F | tacgATTGTACAAGGTACTTCCAA | GAAATGGTCAGCACCGCCAGAAG |
| *lef*-R | aaacTTGGAAGTACCTTGTACAAT | TGTGTCTAATGTAGCAGATACATCTAG |
| GBAA_3660-F | tacgACGACACCTACTCCAAGAGC | AAAATCAGAACATATGTTTCTATTATAG |
| GBAA_3660-R | aaacGCTCTTGGAGTAGGTGTCGT | GAAGATGAAAGAAGATTACGTGAAATTG |

**Table S3.** Safety evaluation groups used in the experiment.

| Number | Immunization route | Antigen | Antigen dose  (μg/mouse) | Adjuvant dose  (μg/mouse) |
| --- | --- | --- | --- | --- |
| 1 | i.t. | CSE（A16R-5.1） | 20 | 20 |
| 2 |  | CSE（A16R-5.1） | 50 | 50 |
| 3 |  | A16R-5 | 20 | 20 |
| 4 |  | A16R-5 | 50 | 50 |
| 5 |  | PBS | / | / |

**Table S4.** Composition and proportion of CSE spray-drying solution

| Composition | Proportion | Properties |
| --- | --- | --- |
| CSE | 0.1% (w/v) | Antigen |
| CpG | 0.1% (w/v) | Antigen |
| D-mannitol | 1% (w/v) | Increases dispersibility |
| Myo-inositol | 1% (w/v) | Enhances stability |
| L-leucine | 0.3% (w/v) | Enhances dispersibility and stability |
| Poloxamer 188 | 0.05% (w/v) | Enhances stability, surfactants |

**Table S5.** Immunization groups of CSE and rPA used in the experiment.

| Number | Immunization route | Antigen | Antigen dose  (μg/mouse) | Adjuvant dose  (μg/mouse) |
| --- | --- | --- | --- | --- |
| 1 | i.t. | CSE | 10 | 10 |
| 2 |  | CSE | 20 | 20 |
| 3 |  | rPA | 10 | 10 |
| 4 |  | rPA | 20 | 20 |

**Table S6.** Identification of top 20 proteins information by LC-MS/MS.

| Sample | Protein IDs | Description | Unique  peptides | Sequence  coverage | iBAQ | LFQ intensity |
| --- | --- | --- | --- | --- | --- | --- |
| A16R | WP_000746486.1 | anthrax toxin protective antigen | 116 | 79.7 | 3581400000 | 5901100000 |
|  | WP_001022097.1 | anthrax toxin lethal factor | 123 | 86.2 | 2743500000 | 5949200000 |
|  | WP_000197748.1 | anthrax toxin edema factor | 99 | 82.5 | 755380000 | 1606600000 |
|  | WP_000476161.1 | NEAT domain-containing protein | 13 | 73.7 | 344360000 | 359790000 |
|  | WP_001080649.1 | S-layer homology domain-containing protein | 40 | 83.3 | 260580000 | 491740000 |
|  | WP_000727239.1 | peptide ABC transporter substrate-binding protein | 49 | 66.3 | 237780000 | 260700000 |
|  | WP_000683998.1 | metal-binding protein ZinT | 16 | 70 | 228070000 | 438880000 |
|  | WP_001140715.1 | S-layer protein EA1 | 78 | 79.6 | 177950000 | 713000000 |
|  | WP_001162675.1 | leucine dehydrogenase | 27 | 68 | 147260000 | 185970000 |
|  | WP_001140751.1 | S-layer protein Sap | 75 | 79.4 | 135660000 | 333390000 |
|  | WP_000670597.1 | DUF5065 family protein | 11 | 67.3 | 130760000 | 136350000 |
|  | WP_000757951.1 | heme uptake protein IsdC | 11 | 47.3 | 127780000 | 285090000 |
|  | WP_000755532.1 | enterotoxin EntFM | 23 | 88.3 | 118750000 | 189260000 |
|  | WP_000850061.1 | lytic polysaccharide monooxygenase | 8 | 29.4 | 104130000 | 284870000 |
|  | WP_000721340.1 | hypothetical protein | 5 | 40 | 102820000 | 173920000 |
|  | WP_000726451.1 | peptide ABC transporter substrate-binding protein | 41 | 65.4 | 102180000 | 326280000 |
|  | WP_000665364.1 | LTA synthase family protein | 27 | 53.4 | 80787000 | 164880000 |
|  | WP_000387561.1 | aureocin A53 family class IId bacteriocin | 134 | 72 | 74333000 | 1325700000 |
|  | WP_000105197.1 | DNA starvation/stationary phase protection protein | 6 | 44.2 | 72236000 | 69803000 |
|  | WP_000746296.1 | S-layer homology domain-containing protein | 35 | 79.1 | 64734000 | 127940000 |
| CSE | WP_000746486.1 | anthrax toxin protective antigen | 123 | 79.7 | 8476900000 | 26121000000 |
|  | WP_000197748.1 | anthrax toxin edema factor | 121 | 82.5 | 2184100000 | 5634800000 |
|  | WP_000476161.1 | NEAT domain-containing protein | 19 | 73.7 | 1368700000 | 1786800000 |
|  | WP_000746296.1 | S-layer homology domain-containing protein | 96 | 79.1 | 1325100000 | 7133600000 |
|  | WP_000755532.1 | enterotoxin EntFM | 31 | 88.3 | 754040000 | 1836700000 |
|  | WP_014654410.1 | cell wall-binding protein EntC | 44 | 77.1 | 626900000 | 3259000000 |
|  | WP_000683998.1 | metal-binding protein ZinT | 17 | 70 | 547130000 | 1822700000 |
|  | WP_000542648.1 | trypsin-like peptidase domain-containing protein | 35 | 70.5 | 479680000 | 1338300000 |
|  | WP_000727131.1 | S-layer homology domain-containing protein | 10 | 39.4 | 445940000 | 1498400000 |
|  | WP_000159736.1 | 50S ribosomal protein L7/L12 | 14 | 87.4 | 346650000 | 522660000 |
|  | WP_000488993.1 | VanW family protein | 22 | 64 | 299130000 | 1122400000 |
|  | WP_001105815.1 | 3D domain-containing protein | 36 | 73.6 | 285600000 | 1503600000 |
|  | WP_000734329.1 | C40 family peptidase | 41 | 75 | 276740000 | 2251600000 |
|  | WP_000665364.1 | LTA synthase family protein | 30 | 53.4 | 238430000 | 1337200000 |
|  | WP_000727239.1 | peptide ABC transporter substrate-binding protein | 53 | 66.3 | 217460000 | 690110000 |
|  | WP_000807831.1 | WXG100 family type VII secretion target | 8 | 74.4 | 215590000 | 526140000 |
|  | WP_001134414.1 | YpjP family protein | 9 | 61.5 | 165960000 | 882490000 |
|  | WP_001162675.1 | leucine dehydrogenase | 28 | 68 | 165170000 | 376170000 |
|  | WP_000662493.1 | signal peptidase I | 13 | 55.7 | 162400000 | 883890000 |
|  | WP_001179150.1 | cold shock-like protein CspB | 4 | 53 | 135610000 | 229960000 |

**Table S7.** Acronym

| Acronym | Full name |
| --- | --- |
| PA | protective antigen |
| AVA | anthrax vaccine adsorbed |
| AVP | anthrax vaccine precipitated |
| LF | lethal factor |
| EF | edema factor |
| LeTx | lethal toxin |
| ET | edema toxin |
| rPA | recombinant PA |
| CpG | CpG oligonucleotide |
| i.t. | aerosolized intratracheal |
| CSE | culture supernatant extract |
| LB | Luria-Bertani |
| BHI | brain heart infusion |
| CRISPR | Clustered Regularly Interspaced Palindromic Repeats |
| PCR | polymerase chain reaction |
| PBS | phosphate-buffered saline |
| SDS-PAGE | sulfate-polyacrylamide gel electrophoresis |
| TMT | tandem mass tags |
| LC-MS/MS | liquid chromatography-tandem mass spectrometry |
| BCA | bicinchoninic acid assay |
| ELISA | enzyme-linked immunosorbent assay |
| SFD | spray freeze drying |
| VMD | volume median diameter |
| MMAD | mass median aerodynamic diameter |
| SPF | specific pathogen-free |
| IACUC | the Institute of Animal Care and Use Committee |
| s.c. | subcutaneous injection |
| BALFs | bronchoalveolar lavage fluids |
| IgG | immunoglobulin G |
| IgG1 | immunoglobulin G1 |
| IgG2a | immunoglobulin G2a |
| SIgA | secretory immunoglobulin A |
| TNA | Toxin-neutralizing antibody |
| IFN-γ | interferon-γ |
| IL-4 | interleukin-4 |
| ELISPOT | enzyme-linked immunospot |
| dppi | post-primary inoculation |
| TSA | tryptic soy agar |
| CFU | colony-forming units |
| PFA | paraformaldehyde |
| H&E | hematoxylin and eosin |
| SD | standard deviation |
| DEPs | differentially expressed proteins |
| iBAQ | intensity-based-absolute-protein-quantification |
| pMDI | Pressurized Metered-Dose Inhalers |
| SMI | Soft Mist Inhalers |
| VLP | Virus-Like Particle |
| SEM | Scanning electron microscopy |
| LoD | Limits of detection |

Supplementary Figures

**Figure S1.** Standard curves of BCA


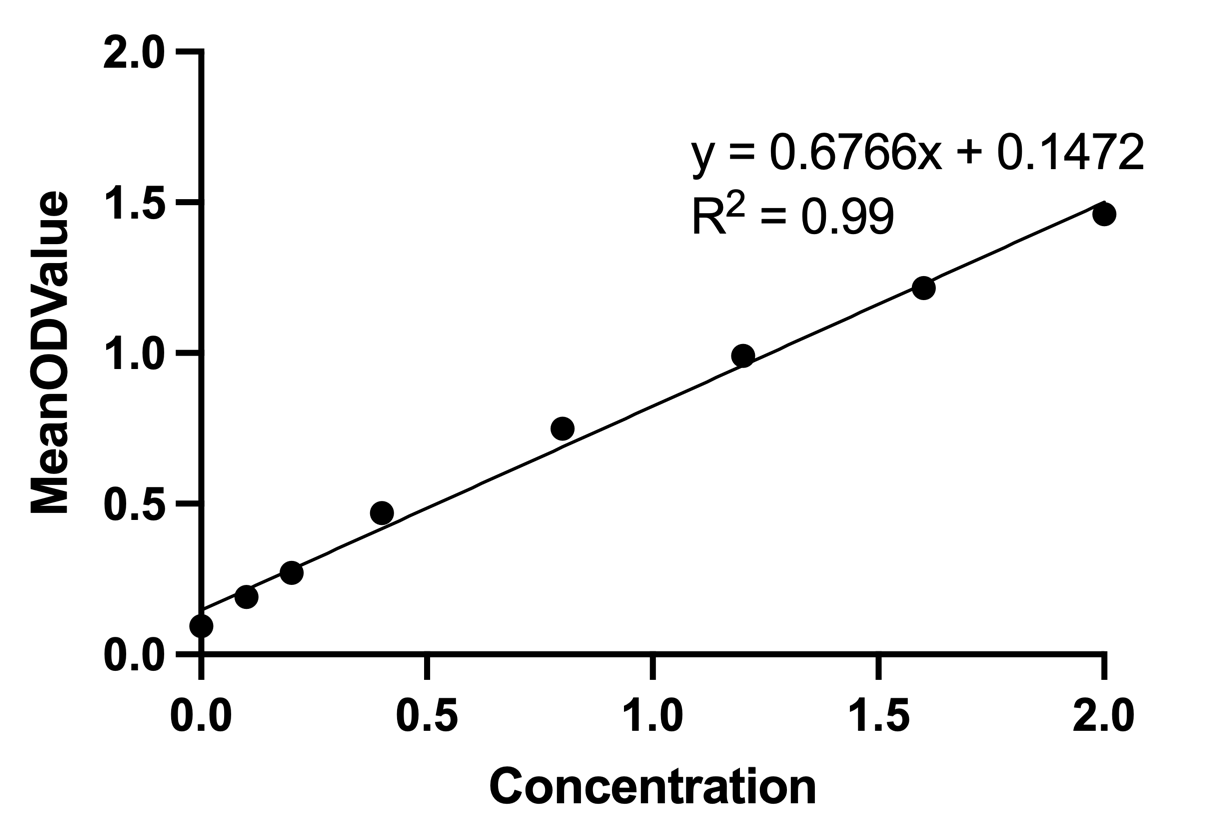


**Figure S2.** Standard curves of ELISA


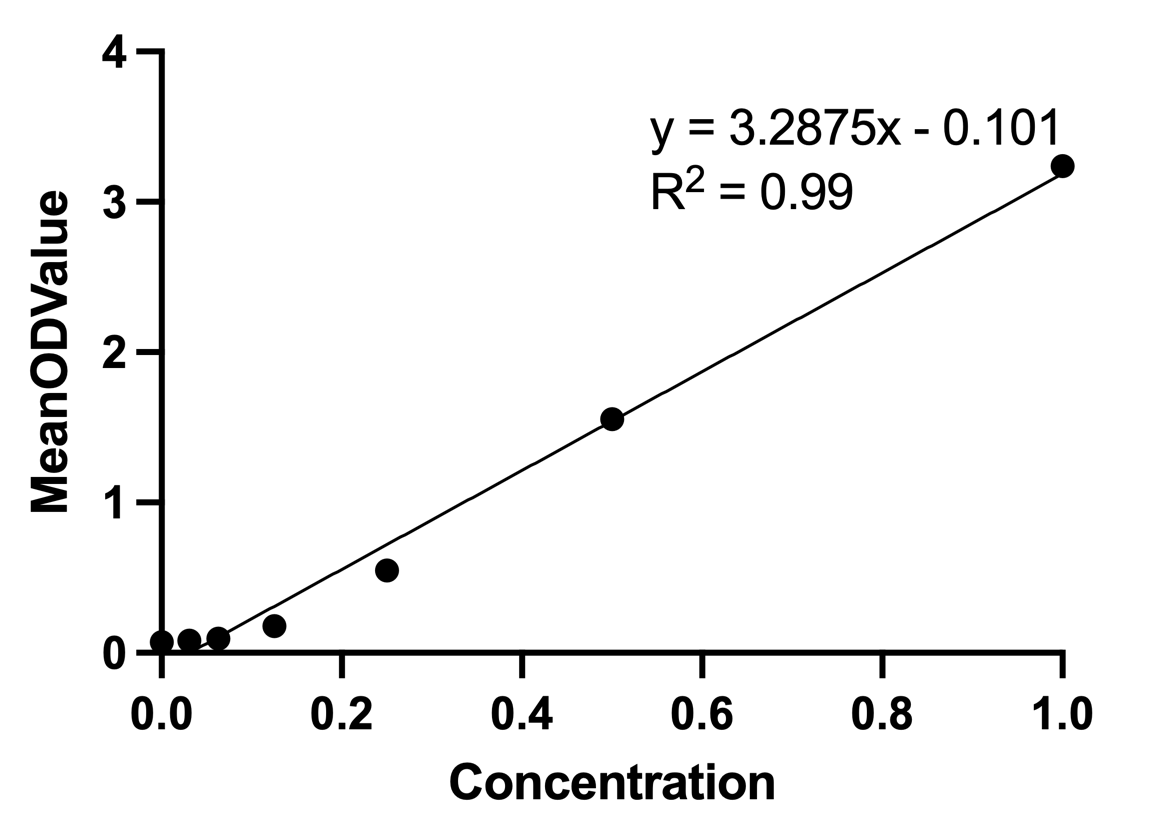


1. Altenbuchner J. Editing of the Bacillus subtilis Genome by the CRISPR-Cas9 System. Appl Environ Microbiol. 2016 Sep 1;82(17):5421-7.
